# Medical Letters from Paris

**Published:** 1853-06

**Authors:** 


MEDICAL LETTERS FROM PARIS.

We publish, in this number, the first of a series of letters from Paris, for which we are indebted to our friend, Prof Gross. Dr. Williams, the writer, is a graduate of the University of Louisville, a young gentleman of fine mind, great application, and genuine love of his profession. "It is exactly six months to-day," he says in a letter to Prof. G., "since I arrived in this capital, and I need not say to you, that I have not been idle. The first three months I devoted almost exclusively to the study of the French language, visiting La Charité, or some other hospital, every other morning, and afterwards listening to the clinical lecture. At the end of three months, I began to understand the lectures very well, and now have no difficulty on that score. I believe there are few persons coming to Paris wholly ignorant of the language who get to understand it in a shorter time than I have done; no one, I am sure, ever devoted himself to the study of it with more obstinate perseverance. I never could imbibe languages or science from the atmosphere; everything I learn comes by dint of hard and untiring study."

Dr. Williams expects to remain twelve or eighteen months longer in Paris, and we hope to lay many of his interesting letters before our readers.

